# Fluoxetine-Induced Changes in *Escherichia coli* Physiology: Effects of Secreted Metabolites on Cell Viability and Colistin Susceptibility

**DOI:** 10.1007/s12602-025-10844-4

**Published:** 2026-01-10

**Authors:** Suna Sibel Rizvanoglu, Murat Sefa Karaaslan, Nuran Gokdere, Aslı Koc, Arzu Zeynep Karabay, Ismail Murat Palabiyik, Nurten Altanlar, Mujde Eryilmaz

**Affiliations:** 1 Department of Pharmaceutical Microbiology, Faculty of Pharmacy, Ankara University, Ankara, Türkiye; 2 Graduate School of Health Sciences, Ankara University, Ankara, Türkiye; 3 Department of Analytical Chemistry, Faculty of Pharmacy, Ankara University, Ankara, Türkiye; 4 Department of Biochemistry, Faculty of Pharmacy, Ankara University, Ankara, Türkiye; 5 Department of Pharmaceutical Microbiology, Faculty of Pharmacy, Acibadem Mehmet Ali Aydinlar University, Istanbul, Türkiye

**Keywords:** Fluoxetine, *Escherichia coli*, Cytotoxicity, HPLC

## Abstract

Fluoxetine has been increasingly recognized for its antimicrobial properties and potential to alter gut microbial composition and function. This study aimed to investigate the effects of fluoxetine exposure on *Escherichia coli* ATCC 25922, focusing on growth curve, protein secretion, metabolite-associated cytotoxicity, antibiotic interaction, and drug depletion. To assess potential impacts on bacterial physiology, *E. coli* was exposed to fluoxetine for 72 h. Bacterial growth and protein levels were measured. Supernatants were tested for cytotoxic effects on SW480 and HMC3 cell lines using MTT assay. Colistin susceptibility of *Pseudomonas aeruginosa* was evaluated in the presence of these supernatants to investigate the indirect modulation of antibiotic susceptibility. Fluoxetine exposure reduced bacterial growth while increasing extracellular protein levels. MIC values for colistin increased over time in both groups but showed no fluoxetine-specific differences. Although cytotoxicity testing specifically found reduced cell viability in fluoxetine-treated culture supernatants, no statistically significant difference was found compared to untreated controls. Finally, HPLC analysis demonstrated complete fluoxetine depletion from the bacterial culture by 72 h. These findings highlight potential microbiota-drug-host interactions and emphasize the need for mechanistic investigation of fluoxetine’s effects on microbial physiology and host cell health.

## Introduction

The gut microbiota is a complex ecosystem that plays a crucial role in human health. Recent studies suggest that it influences not only digestive processes but also neurotransmitter metabolism, central nervous system (CNS) function, and immune responses [1]. The bidirectional communication between the gut microbiota and the CNS, known as the gut-brain axis, has become a focal point in understanding the pathogenesis of neuropsychiatric disorders [2]. Notably, various medications can modulate the composition and function of the gut microbiota, and long-term exposure to these drugs can cause significant changes in microbial diversity, bacterial behavior, and metabolic activity [3–5]. These drugs include statins, laxatives, proton pump inhibitors (PPIs), metformin, beta-blockers, ACE inhibitors, and selective serotonin reuptake inhibitor antidepressants [4].

Selective serotonin reuptake inhibitors (SSRIs), usually prescribed for long-term use, are commonly used in the treatment of psychiatric disorders such as depression. These drugs are known not only for their effects on brain chemistry but also for their potential effects on the gut microbiota [6]. Fluoxetine is one of the most commonly prescribed drugs in this class, used to treat psychiatric disorders such as obsessive-compulsive disorder, major depressive disorder, and anxiety. Recently, fluoxetine has begun to be investigated for its effects on the microbial ecosystem in addition to its effects on human biology [7, 8]. It has also been shown that antidepressants can alter the composition of the gut microbiota and that these changes may influence individuals’ responses to treatment [6]. 

*Escherichia coli* is a facultative anaerobic bacterium commonly found in the human intestine and is therefore frequently used for host-microbe and drug-microbe studies. Studies have shown that exposure of *E. coli* and other gut-associated bacteria to various drugs can alter their growth dynamics, protein secretion, and metabolite production [6–8]. Furthermore, secreted bacterial components can interact with other microorganisms in the intestinal environment or regulate antibiotic susceptibility [9].

Although *Pseudomonas aeruginosa* is not a predominant intestinal species, transient colonization has been reported in immunocompromised or hospitalized patients. It has even been suggested that intestinal colonization may promote the development of infection in the lungs [10, 11]. Due to its clinical importance as an opportunistic pathogen and the critical role of colistin as a last-resort antibiotic, *P. aeruginosa* ATCC 27853 was included in the study to assess whether fluoxetine-exposed *E. coli* supernatants could indirectly affect colistin susceptibility. This will enable us to explore both intra-microbiota effects and interspecies interactions of pharmaceutical products.

Considering these findings, this study aimed to investigate the effects of fluoxetine exposure on *E. coli* ATCC 25922, a common gut-associated bacterium. By exposing *E. coli* to fluoxetine for 72 h, the bacterial growth curve, protein secretion profiles, and the cytotoxic effects of secreted metabolites on human SW480 (colonic epithelial) and HMC3 (microglial) cell lines were examined. To determine whether bacterial metabolites secreted during exposure influence other microorganisms within the intestinal environment, colistin susceptibility was tested in *P. aeruginosa* ATCC 27853, a clinically significant Gram-negative bacterium with intrinsic resistance mechanisms. Colistin was selected as the test antibiotic due to its membrane-disrupting activity, which makes it susceptible to neutralization by outer membrane vesicles (OMVs) or other secreted bacterial components. Additionally, the concentration of fluoxetine in the culture medium was monitored by HPLC to investigate whether the drug was degraded, transformed, or sequestered by the bacteria.

## Materials and Methods

### Biological Materials and Reagents

*E. coli* ATCC 25922 and *P. aeruginosa* ATCC 27853 were used as standard test bacterial strains. Mueller-Hinton Broth (MHB; Merck KGaA, Darmstadt, Germany) and Luria-Bertani (LB) broth (Merck KGaA, Darmstadt, Germany) were used as culture media for minimum inhibitory concentration (MIC) assays. Fluoxetine hydrochloride (Sigma-Aldrich, St. Louis, MO, USA) was used for exposure experiments and prepared in LB broth. Coomassie Brilliant Blue G-250 dye (Merck KGaA, Darmstadt, Germany) was employed in the Bradford assay. High-performance liquid chromatography (HPLC) analysis was conducted using sodium dihydrogen phosphate, methanol, acetonitrile, sodium hydroxide, and phosphoric acid (≥ 99%) (all from Sigma-Aldrich, St. Louis, MO, USA). Chromatographic separation was performed on an ACE 5C18 column (5 μm, 250 mm × 4.6 mm i.d.). An HPLC apparatus (LC2030, Shimadzu, Kyoto, Japan) supplied with a UV detector was utilized. Data handling and acquisition were carried out using Lab Solution software. For cytotoxicity testing, HMC3 cells (human microglial cell line) were cultured in EMEM (Sartorius, Göttingen, Germany), while SW480 cells (human colon cancer cell line) were maintained in RPMI 1640 medium (Gibco, Waltham, MA, USA) supplemented with 10% heat-inactivated fetal bovine serum (Capricorn, Düsseldorf, Germany), 1% L-glutamine (Capricorn, Düsseldorf, Germany), and 1% penicillin-streptomycin (Capricorn, Düsseldorf, Germany). When the cells reached approximately 80% confluency, they were passaged using trypsinization.

### Fluoxetine Sensitivity Test

Before exposure to fluoxetine, the sensitivity of *E. coli* to fluoxetine was determined by the minimum inhibitory concentration (MIC) test. A concentration was determined accordingly for exposure. *E. coli* ATCC 25922 was used as the test bacterium. The MIC test was determined by the microdilution method [12]. Two-fold dilutions of fluoxetine were prepared in MHB medium, and the bacterial suspension prepared from an overnight bacterial culture was inoculated with a final concentration of 5 × 10⁵ CFU/mL in each well. The lowest concentration at which no bacterial growth was observed after 18–24 h of incubation at 35 ± 1 °C was determined as the MIC value.

### Fluoxetine Exposure

The bacterial suspension was prepared by adjusting an overnight *E. coli* culture in LB broth to a density of 0.5 McFarland standard. Then, the bacterial suspension was added to LB broth containing MIC/2 fluoxetine at 1:100 ratio and incubated at 200 rpm and 37 °C for 72 h. LB broth medium inoculated with *E. coli* without fluoxetine was used as a control.

### Bacterial Kinetic Test

Samples were collected at 0, 3, 6, 12, 24, 48, and 72 h, and optical density (OD) was measured at 620 nm using a microplate reader (Thermo Scientific Multiskan GO Microplate Spectrophotometer, Vantaa, Finland). All measurements were performed in triplicate, and bacterial growth curves were generated based on the mean OD values [13].

### Determination of Protein Levels

Protein levels in bacterial supernatants were determined by the Bradford method. The bacterial suspension and culture conditions were as described in Section Fluoxetine Exposure. Supernatants collected at 0, 3, 6, 12, 24, 48, and 72 h were sterilized by filtration using 0.45 μm membrane filters. LB broth medium inoculated with *E. coli* without fluoxetine was used as a blank control. Then, the cell-free supernatants were mixed with Coomassie Brilliant Blue G-250 dye in microplates. The stain gives a blue color when bound to protein. Protein levels were measured at 620 nm using a microplate reader (Thermo Scientific Multiskan GO Microplate Spectrophotometer, Vantaa, Finland) [13]. Experiments were performed with two independent biological replicates. Each biological replicate was measured in eight technical replicates for each condition and time point. A two-way ANOVA with replication was used to evaluate changes between the control and fluoxetine groups at various time points for protein measurements. The analysis included two independent variables: treatment (control-MIC/2 fluoxetine) and time (0, 3, 6, 12, 24, 48, and 72 h). The interaction effect between these factors was also examined. When significant differences emerged, the Tukey HSD post-hoc test was applied to evaluate multiple group comparisons. Statistical significance was accepted at *p* < 0.05.

### Determination of Colistin Minimum Inhibitory Concentration in the Presence of Fluoxetine-Exposed Secreted Proteins

To investigate whether extracellular proteins secreted by *E. coli* after 72 h of fluoxetine exposure affect the MIC value of colistin against *P. aeruginosa* ATCC 27853, the MIC value was determined by the microdilution method in the presence of bacterial supernatants. Colistin was serially diluted two-fold in the collected supernatants. Then, *P. aeruginosa* bacterial suspensions were added to each well at a final concentration of 5 × 10⁵ CFU/mL. Microdilution plates were then incubated at 35 ± 1 °C for 18–24 h according to the broth microdilution protocol recommended by the European Committee on Antimicrobial Susceptibility Testing (EUCAST) [12]. After incubation, the wells were examined, and the lowest concentration at which no visible bacterial growth occurred was recorded as the MIC value.

### Determination of Fluoxetine Concentration

To prepare standard solutions of fluoxetine, 10.0 mg of fluoxetine was weighed and completed to 100 mL with methanol. To prepare different concentrations, the standard solution was diluted with ultrapure water. Stored in the refrigerator when stored at 4 °C. The method developed by Magdy et al. [14] was modified for quantification in HPLC. Sodium dihydrogen phosphate buffer: acetonitrile (7.5 mM; pH 4; 40: 60% v/v) was used as the mobile phase. A reversed-phase C18 column (5 μm particle size, 250 mm × 4.6 mm inner diameter) was used for the stationary phase. Isocratic elution was performed with a flow rate of 1.50 mL/min and an injectable volume of 30.0 µL. The maximum wavelength for the UV detector was 235 nm. The retention time of fluoxetine was found to be 2.675 min. 7 samples ranging from 0.5 to 30 ppm concentration were prepared in 3 series and analyzed under optimum chromatographic conditions. Analyses were repeated twice. Limit of detection (LOD) and limit of quantification (LOQ) were calculated using the following formulas:

1 $$\:LOD=3.3\times\:\sigma/m$$

2 $$\:LOQ=10\times\:\sigma/m$$

### Cytotoxic Activity Test

#### Cell Viability

Cell viability was assessed using the MTT assay (3-(4,5-dimethylthiazol-2-yl)−2,5-diphenyltetrazolium bromide). For this purpose, 10,000 cells per well were seeded into 96-well plates. The control and experimental groups used in the study are summarized in Table 1. After overnight incubation to allow adherence, bacterial metabolites were applied at concentrations of 0.5% and 1%. SW480 and HMC3 cells were then incubated with the supernatants for 72 h. Following treatment, a 5 mg/mL MTT solution prepared in PBS was added to the wells. After 4 h of incubation, formazan crystals were dissolved using an SDS-HCl solution. Absorbance was measured at 550 nm using a microplate reader. The medium in which the bacteria were cultured (B) served as the control group and was considered to represent 100% cell viability. No statistically significant difference in viability was observed between cells treated with RPMI or EMEM medium alone (C) and those treated with the bacterial culture medium.

**Table 1:** Control and experimental groups in the study

K0	Cell-free supernatant collected at 0 h from fluoxetine-untreated cultures
K24	Cell-free supernatant collected at 24 h from fluoxetine-untreated cultures
K48	Cell-free supernatant collected at 48 h from fluoxetine-untreated cultures
K72	Cell-free supernatant collected at 72 h from fluoxetine-untreated cultures
M0	Cell-free supernatant collected at 0 h from fluoxetine-treated cultures
M24	Cell-free supernatant collected at 24 h from fluoxetine-treated cultures
M48	Cell-free supernatant collected at 48 h from fluoxetine-treated cultures
M72	Cell-free supernatant collected at 72 h from fluoxetine-treated cultures
A24	Fluoxetine-containing culture medium collected after 24 h of incubation in the absence of bacteria
A48	Fluoxetine-containing culture medium collected after 48 h of incubation in the absence of bacteria
A72	Fluoxetine-containing culture medium collected after 72 h of incubation in the absence of bacteria
B	LB broth
C	Cells incubated with RPMI or EMEM medium used in cell culture

#### Statistical Analysis

Statistical analyses were performed using GraphPad Prism 9. The Shapiro-Wilk test was used to assess the normality of data distribution. Differences between groups were evaluated using the Kruskal-Wallis test. A p-value of < 0.05 was considered statistically significant. Data are presented as mean ± standard deviation.

## Results

### Result of Fluoxetine Sensitivity Test

As a result of the microdilution method, the MIC value of Fluoxetine against *E. coli* ATCC 25922 was found to be 31.25 µg/mL. Exposure concentration was prepared according to the determined MIC value.

### Bacterial Growth Curve Under Fluoxetine Exposure

**Fig. 1:**
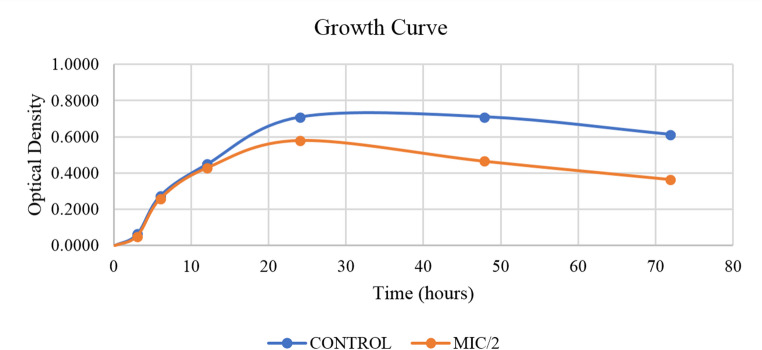
Growth curve of *Escherichia coli *ATCC 25922 exposed to fluoxetine. The growth curve of *E. coli* was monitored over a 72-hour incubation period in Luria-Bertani (LB) broth with and without MIC/2 fluoxetine. Control cultures (blue line) were incubated in LB broth alone, while treated cultures (orange line) contained fluoxetine. Optical density (OD) values at 620 nm were recorded at 0, 3, 6, 12, 24, 48, and 72 hours. While both groups followed a similar growth pattern (lag, exponential, and stationary phases), fluoxetine exposure resulted in a consistently lower OD, indicating growth inhibition rather than complete suppression. These results suggest that sub-inhibitory concentrations of fluoxetine may interfere with bacterial proliferation

The 72-hour growth dynamics of *E. coli* were examined in LB broth with MIC/2 fluoxetine. Control culture was incubated in LB broth without fluoxetine under identical conditions. Samples were collected at 0, 3, 6, 12, 24, 48, and 72 h. The samples were measured at 620 nm, and the growth curve of the bacteria was presented in Fig. 1.

*E. coli* exposed to fluoxetine at the MIC/2 concentration exhibited reduced growth compared to the control group. However, the overall growth curve pattern remained similar between the treated and control groups.

### Determination of Total Protein Levels after Exposure

**Fig. 2:**
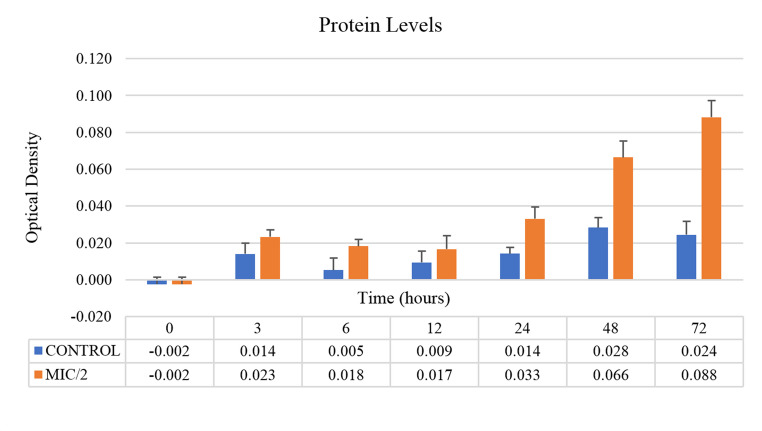
Comparative protein levels in control and fluoxetine-treated *E. coli* supernatants over 72 hours. Statistical analysis by two-way ANOVA revealed significant effects of treatment (*p*<0.0001), time (*p*<0.0001), and treatment/time interaction (*p*<0.0001). Tukey’s post-hoc test indicated significantly higher protein levels in the fluoxetine group at 3, 6, 24, 48, and 72 h compared with controls (*p*<0.05), while no significant differences were observed at 0 and 12 h

Protein levels of supernatants taken from all test conditions were calculated using the Bradford method using absorbance values ​​measured in the spectrometer (Fig. 2).

Following fluoxetine exposure, a decrease in bacterial growth was observed compared to the control group. However, the amount of extracellular proteins secreted by the bacteria was higher than that of the control group. This finding suggests that fluoxetine exposure may induce cellular stress, leading to increased secretion of extracellular proteins or potential alterations in the composition of the secreted protein profile. According to the statistical evaluation, two-way ANOVA showed that fluoxetine treatment (*p* < 0.0001), time (*p* < 0.0001), and their interaction (*p* < 0.0001) were statistically significant. Tukey’s post-hoc analysis revealed that protein secretion levels were significantly increased in the fluoxetine-treated group compared to the control at 3, 6, 24, 48, and 72 h (*p* < 0.05), whereas no significant difference at 0 and 12 h. These findings suggest that fluoxetine exposure triggered a time-dependent increase in protein secretion by *E. coli*, especially from the 24th hour onwards.

### Colistin MIC Result

To evaluate the potential impact of secreted components present in the supernatants of fluoxetine-treated and control *E. coli* cultures on colistin activity, MIC assays were conducted against *P. aeruginosa* ATCC 27853 using these supernatants as growth media (Table 2).

**Table 2:** MIC values of colistin against *P. aeruginosa* in the presence of *E. coli* supernatants (µg/mL)

Time points (hours)	Control Supernatant	Fluoxetine-Treated Supernatant
**0**	0.25	0.5
**3**	0.5	1
**6**	2	2
**12**	4	2
**24**	4	4
**48**	4	4
**72**	4	4

A gradual increase (~ 16-fold) in the MIC value of colistin was observed over time in both supernatants collected at different incubation time points. A rapid increase in MIC was observed during the first 6 h in both groups and reached 2 µg/mL. However, from 12th hour supernatant onwards, the MIC values ​​stabilized at 4 µg/mL in both groups and remained unchanged until 72nd hour supernatant.

### High-Performance Liquid Chromatography (HPLC) Analysis

**Fig. 3:**
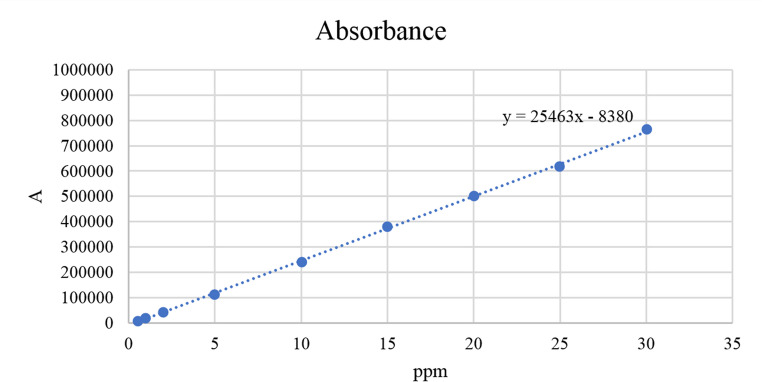
Calibration curve of fluoxetine

HPLC was performed to monitor the concentration of fluoxetine in the culture medium over time. The calibration curve for fluoxetine analysis is given in Fig. 3. Analysis parameters of the analytical method used are presented in Table 3.

**Table 3:** Evaluation table of the analytical method used

Parameters	Result
Linear dynamic range (ppm)	0.5 –30
Correlation coefficient (R^2^)	0.99
LOD (ppm)	0.14
LOQ (ppm)	0.43
Repeatability (%RSD)	1.32
Accuracy (% Recovery)	98.28–101.93

**Fig. 4:**
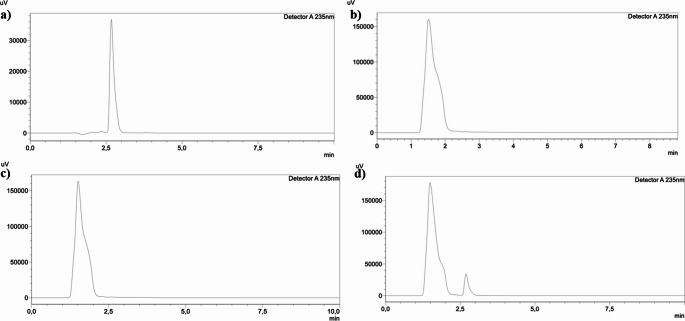
Chromatograms of **a**) fluoxetine standard solution, **b**) medium (LB broth), **c**) control (K), and **d**) fluoxetine with medium (M). Analyses were carried out using an HPLC-UV system. Column: C18 (250 × 4.6 mm, 5 µm); mobile phase: sodium dihydrogen phosphate buffer: acetonitrile (7.5 mM; pH 4; 40:60, v/v); flow rate: 1.50 mL·min⁻¹; injection volume: 30 µL; detection wavelength: 235 nm

The results of the HPLC analysis of fluoxetine are presented in Table 4. No fluoxetine was detected in the Control (Medium + Bacteria) at any time point. In contrast, fluoxetine remained detectable in both the A (Medium + Fluoxetine) and the M (Medium + Bacteria + MIC/2 Fluoxetine). By the end of the 72 h, fluoxetine levels had declined in both samples; however, while it remained detectable in the A, it was completely depleted in the M. HPLC chromatograms are shown in Fig. 4. The medium and control were analyzed for selectivity. The absence of any peaks in the medium and control groups, where fluoxetine peaks in the chromatograms, indicates that this study was highly selective.

**Table 4:** Time-Dependent changes in Fluoxetine concentration (ppm) in samples

Sample Group	Time Point (h)	Fluoxetine Concentration (ppm)
A (Medium + MIC/2 Fluoxetine)	0	17.8
	3	15.5
	6	11.5
	12	11.2
	24	11.1
	48	10.8
	72	7.6
K (Medium + Bacteria)	All time points	-
M (Medium + MIC/2 Fluoxetine + Bacteria)	0	17.9
	3	15.9
	6	14.5
	12	13.0
	24	11.2
	48	10.1
	72	-

“–” indicates that no fluoxetine peak was detected.

### Cytotoxic Activity Test

The effects of bacterial culture supernatants were compared between cells treated with K0 and those treated with K24, K48, and K72, as well as with cells exposed to the bacterial growth medium (B). The effects of fluoxetine-exposed supernatants were evaluated by comparing cells treated with supernatants collected from fluoxetine-treated bacterial cultures to those treated with: (1) medium containing the same concentration of fluoxetine and incubated under the same conditions, and (2) the supernatants collected from fluoxetine-treated bacterial cultures at 0 h (M0).

**Fig. 5:**
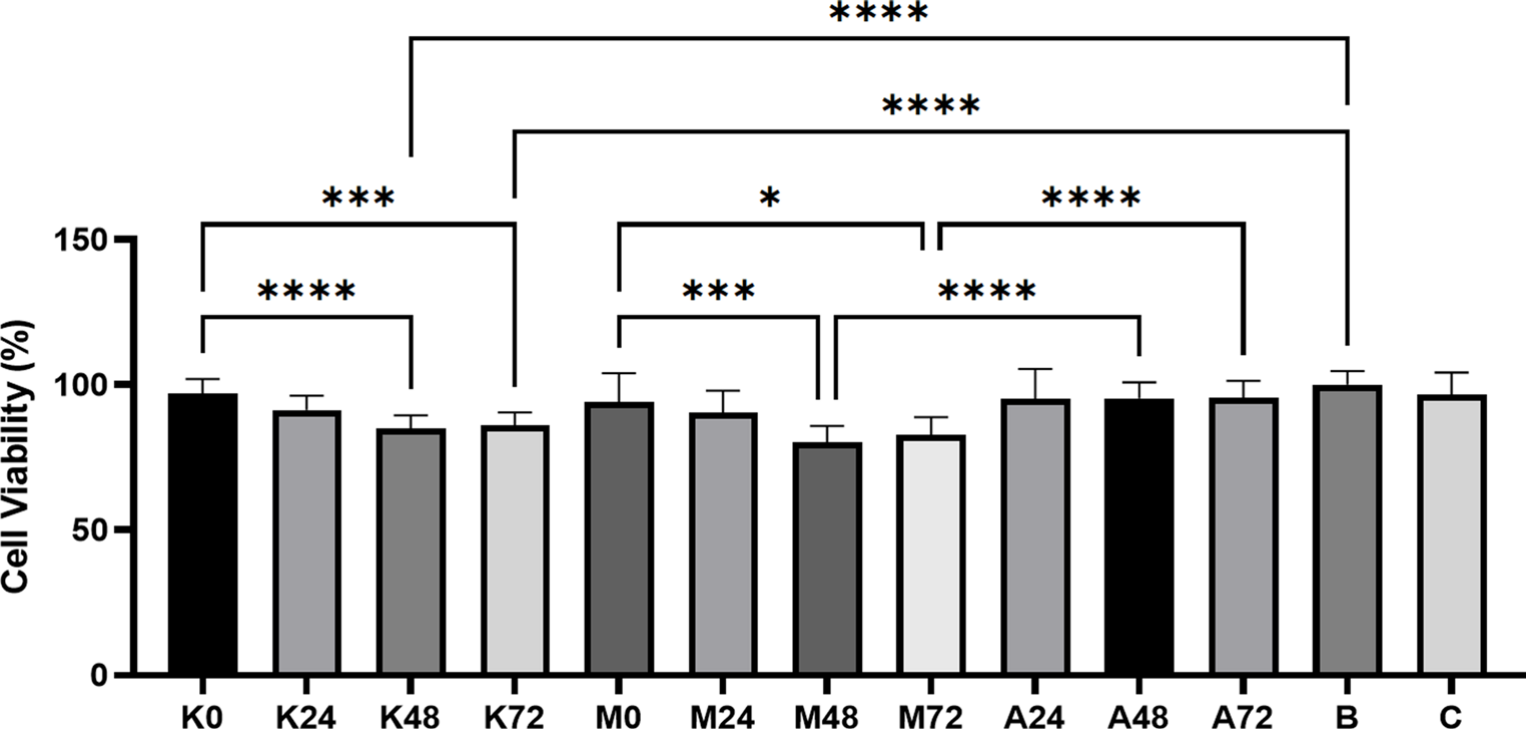
Cell viability (%) of SW480 cells treated with 0.5% concentrations of bacterial supernatants for 72 h (*p < 0.05; **p < 0.01; ***p < 0.001; ****p < 0.0001). What K0, K24, K48, K72, M0, M24, M48, M72, A24, A48, A72, B and C represent is shown in Table 1. Data are given as mean ± standard deviation. (*n* = 21)

In SW480 cells incubated with 0.5% bacterial supernatants for 72 h, the K48 and K72 samples showed a significant decrease in cell viability compared to K0 and as well as compared to B. M48 and M72 significantly reduced cell viability compared to both A48 and A72, as well as compared to M0. When we compared the effects of supernatants from untreated cultures (K24, K48, and K72) with those from fluoxetine-treated cultures (M24, M48, and M72), no statistically significant difference was found between them. Among all groups, M48 induced a slightly greater reduction in cell viability compared to the other groups. (Fig. 5; Table 5).

**Table 5:** Cell viability (%) of SW480 and HMC3 cells treated with 0.5% and 1% concentrations of bacterial supernatants

	SW480 0.5%	SW480 1%	HMC3 0.5%	HMC3 1%
**K0**	96.86 ± 5.08	104.5 ± 3.64	103.3 ± 4.75	102.2 ± 5.63
**K24**	91.37 ± 4.81	94.04 ± 2.88	95.63 ± 3.9	88.30 ± 5.487
**K48**	84.94 ± 4.47	85.47 ± 3.88	93.79 ± 2.86	87.5 ± 6.13
**K72**	85.88 ± 4.61	85.01 ± 3.90	93.41 ± 4.160	89.45 ± 4.25
**M0**	93.92 ± 10.03	100.9 ± 7.27	101.3 ± 4.59	96.78 ± 3.51
**M24**	90.49 ± 7.411	90.00 ± 2.53	93.6 ± 3.48	86.89 ± 6.12
**M48**	80.17 ± 5.64	85.59 ± 4.94	90.24 ± 5.47	87.73 ± 6.015
**M72**	82.68 ± 6.09	84.64 ± 3.52	91.60 ± 2.17	86.60 ± 3.99
**A24**	95.12 ± 10.29	97.79 ± 7.19	99.87 ± 5.7	98.23 ± 7.30
**A48**	95.35 ± 5.52	96.97 ± 5.76	99.95 ± 5.73	97.69 ± 3.86
**A72**	95.56 ± 5.75	96.30 ± 3.82	99.58 ± 7.28	97.91 ± 2.76
**B**	100 ± 4.68	100 ± 3.24	100 ± 10.37	100 ± 3.14
**C**	96.61 ± 7.59	100.6 ± 6.87	99.40 ± 3.19	97.98 ± 3.235

**Fig. 6:**
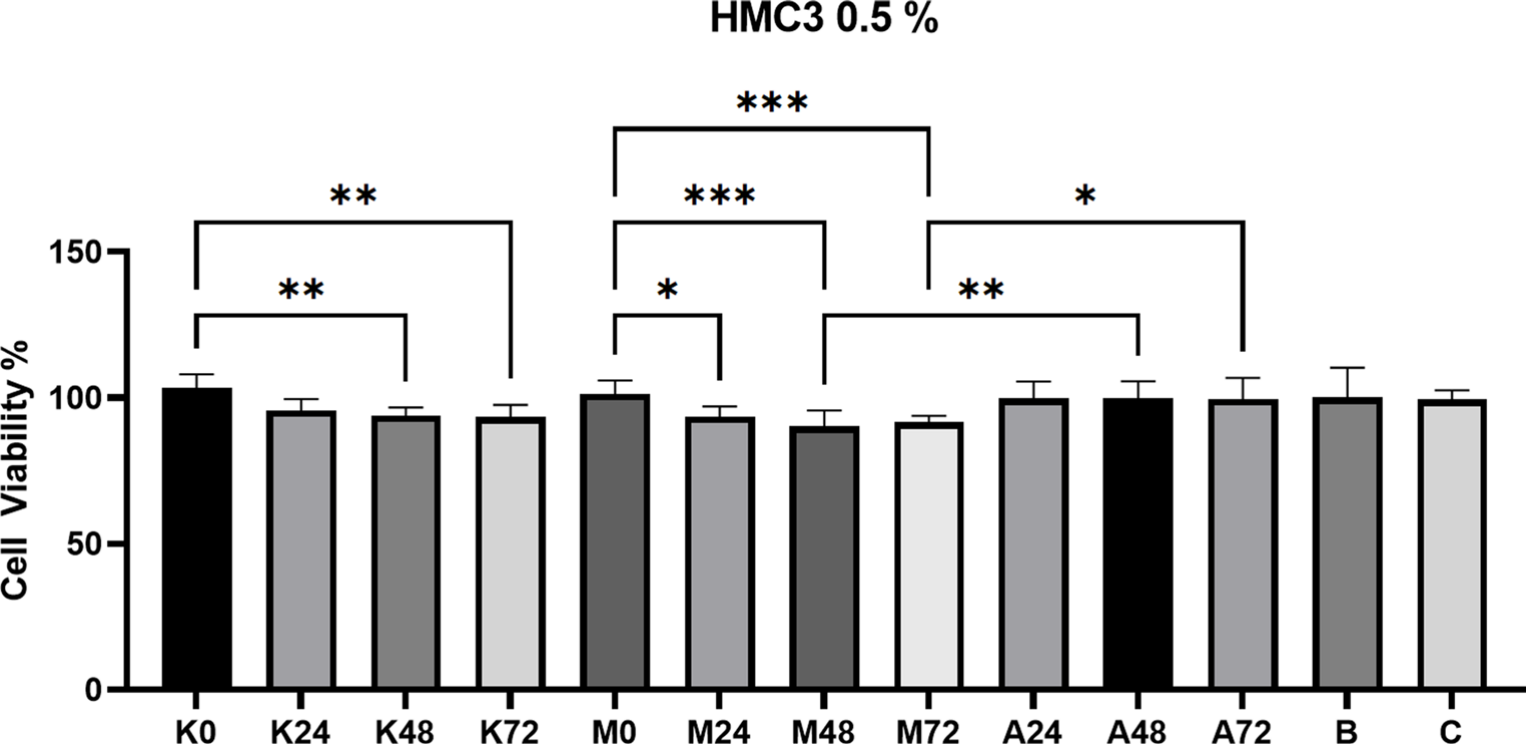
Cell viability (%) of HMC3 cells treated with 0.5% concentrations of bacterial supernatants for 72 h (*p < 0.05; **p < 0.01; ***p < 0.001; ****p < 0.0001). What K0, K24, K48, K72, M0, M24, M48, M72, A24, A48, A72, B and C represent is shown in Table 1. Data are given as mean ± standard deviation. (*n* = 14)

In HMC3 cells incubated with 0.5% bacterial supernatants, K48 and K72 significantly reduced cell viability compared to K0. However, no significant change was observed between K24, K48 and K72 with HMC3 cells treated with medium B, in which only bacteria were grown. Additionally, M24, M48 and M72 showed decreased cell viability compared to M0. Furthermore, both M48 and M72 significantly reduced viability compared to A48 and A72. However, in the HMC3 cell line, no significant differences were observed between K24, K48, and K72 and M24, M48, and M72 when applied at a 0.5% concentration. M48 induced a slightly greater reduction in cell viability compared to the other groups. (Fig. 6; Table 5).

**Fig. 7:**
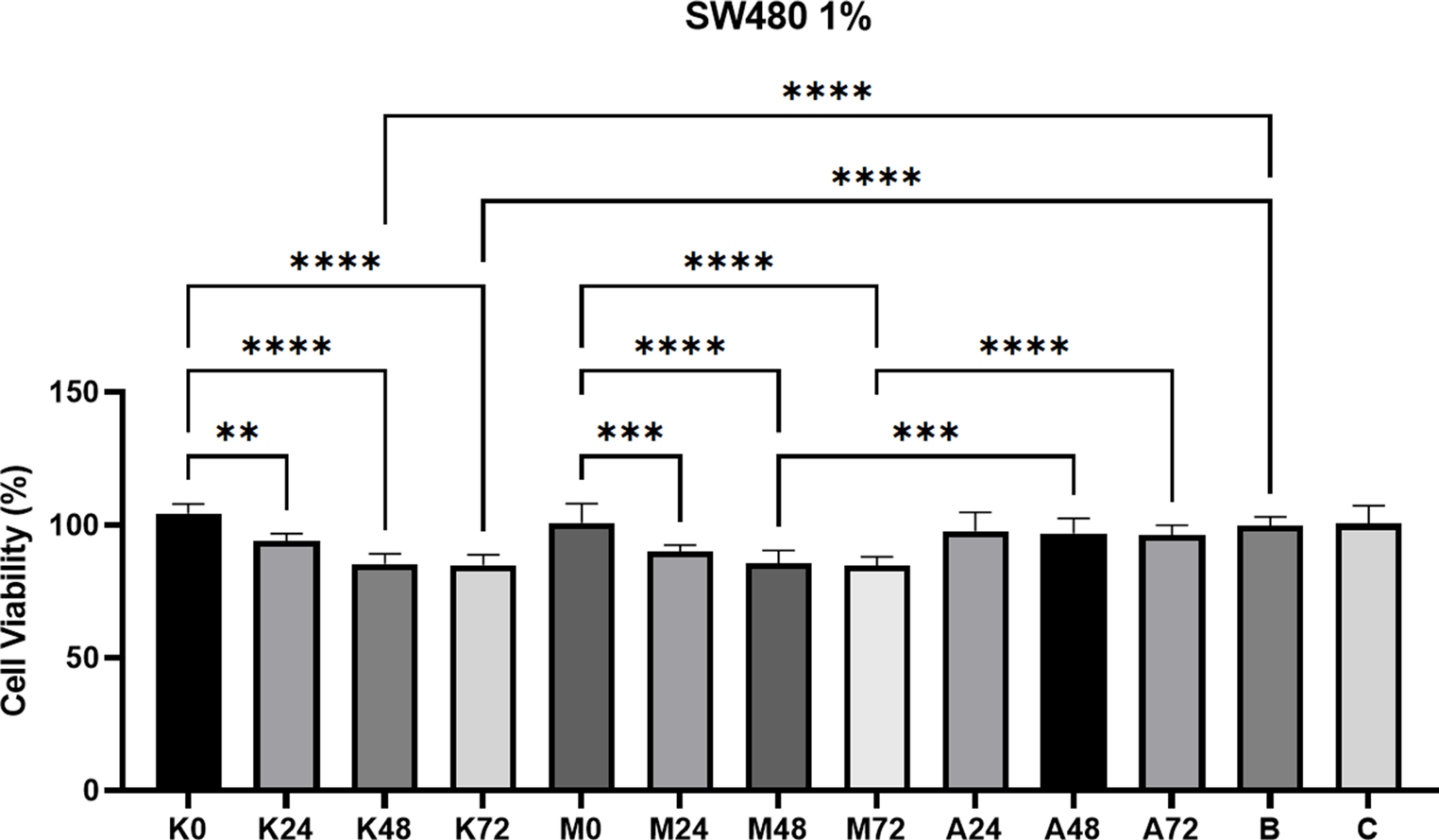
Cell viability (%) of SW480 cells treated with 1% concentrations of bacterial supernatants for 72 h (*p < 0.05; **p < 0.01; ***p < 0.001; ****p < 0.0001). What K0, K24, K48, K72, M0, M24, M48, M72, A24, A48, A72, B and C represent is shown in Table 1. Data are given as mean ± standard deviation. (*n* = 21)

SW480 cells incubated with 1% bacterial supernatants for 72 h, K24, K48, and K72 samples significantly decreased cell viability compared to K0. A significant difference was found between the viability of cells treated with the medium in which bacteria were grown (B) and K48 or K72. Additionally, M24, M48, and M72 showed decreased cell viability compared to M0. Furthermore, both M48 and M72 significantly reduced viability compared to A48 and A72. However, in the SW480 cell line, no significant differences were observed between K24, K48, and K72 and M24, M48, and M72 when applied at a 1% concentration. Among all groups, M72 exhibited the most pronounced decrease in cell viability (Fig. 7; Table 5).

In HMC3 cells, 1% bacterial supernatants, K24, K48, and K72 samples significantly decreased cell viability compared to K0. A significant difference was found between the viability of cells treated with the medium in which bacteria were grown (B) and K24, K48, and K72. Supernatants collected from fluoxetine-treated bacterial cultures (M24, M48, and M72) also reduced cell viability compared to both the fluoxetine-containing medium alone (A24, A48, A72) and the M0 group. Statistically significant differences were observed between M0 and both M24 and M72, as well as between A24 and M24, and A72 and M72.

**Fig. 8:**
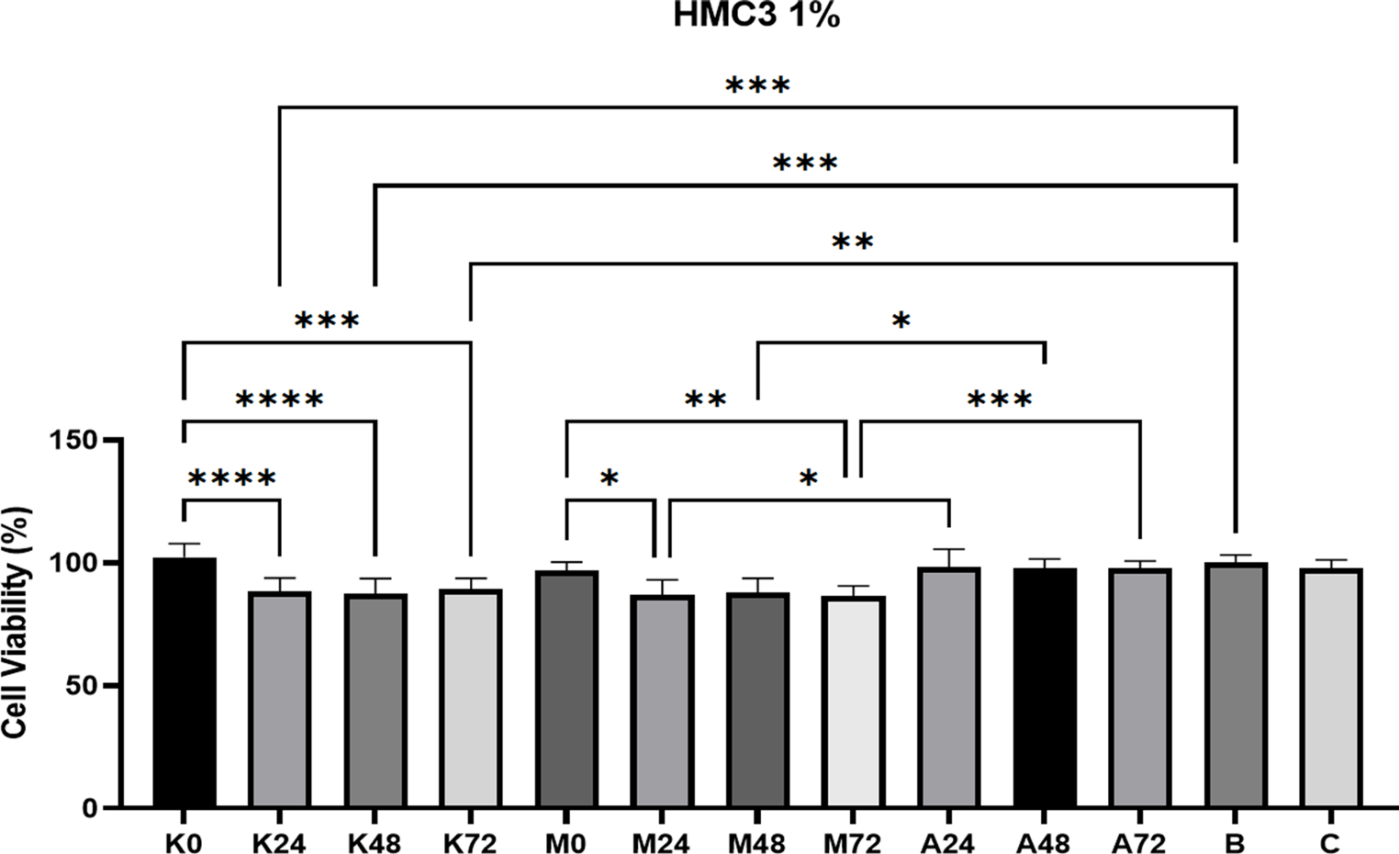
Cell viability (%) of HMC3 cells treated with 1% concentrations of bacterial supernatants for 72 h (*p < 0.05; **p < 0.01; ***p < 0.001; ****p < 0.0001). What K0, K24, K48, K72, M0, M24, M48, M72, A24, A48, A72, B and C represent is shown in Table 1. Data are given as mean ± standard deviation. (*n* = 13)

When the effects of bacterial supernatants (K24, K48, and K72) produced under standard growth conditions without fluoxetine were compared with those of supernatants (M24, M48, and M72) derived from fluoxetine-treated cultures on the viability of HMC3 cells, no statistically significant differences were observed between the groups. However, M72 induced a slightly greater reduction in cell viability compared to the other groups. (Fig. 8; Table 5).

## Discussion

Recent studies have shown that pharmaceutical compounds act not only at targeted sites but also on the composition and function of the gut microbiota. Drugs may alter the intestinal microenvironment and microbial metabolism or affect bacterial growth, thereby changing microbial community composition and function. SSRIs, such as fluoxetine, have been shown to alter microbial diversity, bacterial metabolism, and host-microbiota interactions [6, 8, 15]. 

*E. coli*, a natural part of the intestinal microbiota, plays an important role in host metabolic balance, immune modulation, and maintenance of intestinal barrier function [16]. Studies have shown that fluoxetine can disrupt beneficial interactions with the host by affecting the growth patterns and protein secretion profiles of *E. coli*. These effects can result in serious consequences, such as the development of antibiotic resistance and disruption of the intestinal microbiota balance [17–19]. Such disruptions may cause various adverse effects, including increased intestinal permeability, altered short-chain fatty acid production, increased inflammatory responses, and disturbances in the gut-brain axis, which may paradoxically worsen neuropsychiatric symptoms [1, 5, 6]. In Gram-negative bacteria, factors such as environmental stress, antibiotics, or host immunity can lead to increased production of outer membrane vesicles (OMVs) [20, 21]. OMVs serve various functions, including transporting virulence factors, modulating the immune response, transferring genetic material, facilitating microbial communication, and influencing community dynamics. They also help protect bacteria against certain antibiotics [20]. Structurally, these vesicles resemble the bacteria’s outer membrane and may contain lipopolysaccharide (LPS), outer membrane proteins, phospholipids, and even enzymes that inactivate antibiotics [9, 22]. OMVs can act as decoys, providing a protective shield against antibiotics. In particular, membrane-targeted antibiotics like colistin can bind to the OMVs instead of the bacteria, thus contributing to protecting the bacteria against antibiotics [20].

Based on all these data, an *E. coli* standard strain was exposed to fluoxetine for 72 h to investigate bacterial growth curve, protein secretion profiles, and the potential effect of secreted compounds against SW480 and HMC3 cell lines and colistin resistance of *P. aeruginosa*.

Many studies have shown that fluoxetine has antimicrobial properties [17, 23]. In our study, the MIC value of fluoxetine against *E. coli* ATCC 25922 was determined as 31.25 µg/mL. To explore sublethal effects, growth curve and extracellular protein secretion levels were evaluated at the MIC/2 concentration. Due to its antimicrobial activity, fluoxetine can alter bacterial growth dynamics and affect basic physiological processes [7, 17]. Similar to our findings, Rukavishnikov et al. [19] have reported that fluoxetine significantly reduced the growth activity of *E. coli* ATCC 25922. Another study evaluating the antimicrobial effects of psychotropic drugs on members of the human gut microbiota, such as *E. coli* and *Lactobacillus rhamnosus*, showed that fluoxetine and escitalopram inhibited the growth of these bacteria. Additionally, in experiments conducted on rats, fluoxetine treatment altered the diversity and composition of the gut microbiota. In particular, increased intestinal permeability was observed in the ileum of animals treated with fluoxetine and escitalopram [6]. This suggests that fluoxetine may have biological effects not only on the host’s nervous system but also on the gut microbiota.

In our study, the growth curve obtained after 72 h of exposure to fluoxetine at the MIC/2 concentration revealed that bacterial proliferation was reduced compared to the control group. However, total protein levels measured in the supernatants at the same time points were higher in the MIC/2 fluoxetine- treated group and reached approximately a fourfold increase by 72 h. It is well established that exposure to fluoxetine induces oxidative stress in bacteria through the generation of reactive oxygen species (ROS), which may potentially activate stress-related pathways, including increased OMVs release [17, 20, 24]. Although higher extracellular protein levels were observed after sub-MIC fluoxetine exposure in our study, Bradford-based assays cannot exclude contributions from cell damage. To minimize this effect, sub-MIC fluoxetine was used to avoid bactericidal activity, and control cultures were included to account for protein release due to cell damage. Nonetheless, complementary analyses, such as viability testing, OMV isolation, and proteomic characterization, would provide a more detailed understanding of the molecular origin and functional significance of the observed extracellular proteins. Moreover, OMVs, which structurally resemble the bacterial outer membrane, can interact with membrane-targeting antibiotics like colistin, sequestering them and thereby reducing their antibacterial efficacy [25]. Based on these findings, we hypothesized that secreted components, present in the supernatants of fluoxetine- treated *E. coli* cultures, may interfere with colistin activity. To test this, we used supernatants collected after 72 h of incubation from both fluoxetine-treated and control *E. coli* cultures as the growth medium for *P. aeruginosa* ATCC 27853, and determined the MIC values for colistin. *P. aeruginosa* was included in this study because it represents a clinically relevant, well-characterized Gram-negative pathogen frequently encountered in hospital and polymicrobial environments, including those where *E. coli* may also be present. This approach provided a controlled context to test whether fluoxetine-exposed *E. coli* metabolites could influence colistin susceptibility. Notably, an increase in MIC was observed in both groups compared to the baseline. The final stabilization of MIC values in both groups highlights the potential role of time-dependent accumulation of extracellular factors, but does not support a lasting or fluoxetine-specific modulatory effect. Proteomic analyses will be valuable for further characterizing the molecular components underlying this phenomenon. Expanding future investigations to include antibiotics with distinct mechanisms of action could help clarify whether fluoxetine-induced secretion dynamics selectively affect membrane-active compounds or have wider implications for bacterial stress responses.

The HPLC method developed in this study enabled accurate and reliable determination of fluoxetine concentration in culture media. The method’s low limit of detection (LOD: 0.14 ppm) and wide linear range (0.5–30 ppm) allowed for precise monitoring of fluoxetine changes over time in microbial environments. Furthermore, high recovery percentages (98.28–101.93%) and low relative standard deviations (below 2%) demonstrate the method’s reproducibility and accuracy. In A (medium + fluoxetine), the fluoxetine concentration has decreased over 72 h, reaching approximately 7.6 ppm at the end of the incubation. This decrease can be attributed to physicochemical factors such as chemical instability, light exposure, and interaction with medium components. Lam et al. [26] have reported that fluoxetine photodegrades in aqueous solutions under light with a half-life of approximately 55.2 ± 3.6 h, supporting our observation of a gradual decrease. In M (medium + fluoxetine + bacteria), fluoxetine levels became undetectable within 72 h. This contrast suggests a bacteria-dependent mechanism of fluoxetine depletion. Several studies have reported that bacteria can metabolize or biotransform pharmaceutical agents, including fluoxetine, or facilitate their intracellular accumulation [18, 27, 28]. Xu et al. [18], for instance, demonstrated that antidepressants can not only alter microbial composition but also affect microbial enzymatic pathways, contributing to the breakdown of active compounds. In addition to metabolism, fluoxetine disappearance may be partially explained by sequestration via bacterial OMVs. Although this mechanism has not yet been directly demonstrated for fluoxetine, its lipophilic structure makes such interactions with the lipid components of OMVs plausible. These vesicles have structural similarities with the bacterial outer membrane and are known to interact with hydrophobic or membrane-active molecules [20, 25]. The lack of detectable fluoxetine peaks after 72 h in the M may be due to all these factors. Zhang et al. [29] have shown that fluoxetine can significantly alter the intestinal microbial composition and bacterial metabolic pathways. Thus, fluoxetine-exposed *E. coli* might exhibit an altered secretion profile, potentially including increased OMV release, which could contribute to the removal of fluoxetine from the culture medium. Our findings indicate that fluoxetine depletion accelerated between 48 and 72 h. Future research, including additional intermediate sampling points, could help clarify the relative contributions of bacterial metabolism, OMV sequestration, and physicochemical degradation to fluoxetine depletion.

The intestinal microbiota plays a role in maintaining host health by actively participating in fundamental physiological processes such as metabolic balance, immune regulation, and epithelial barrier integrity [30, 31]. Recent studies have shown that non-antibiotic drugs such as antidepressants can also directly affect the microbiota, and that such drugs suppress the growth of some bacterial species, alter their protein secretions, and indirectly affect metabolite profiles [6, 7]. Therefore, evaluating pharmacological agents not only from a pharmacokinetic perspective but also from a pharmacomicrobiomic perspective will provide a better understanding of their systemic effects and interindividual variability.

To examine the potential effect of drug-microbiota interactions on host cell viability, cytotoxicity assays were conducted using supernatants from fluoxetine-treated and untreated bacterial cultures, and control medium containing only fluoxetine at the same concentration. In both SW480 colorectal cancer cells and HMC3 microglial cells, a reduction in cell viability was observed following exposure to bacterial supernatants, those derived from fluoxetine-treated cultures. In contrast, the fluoxetine-containing media without bacterial processing induced less pronounced effects, suggesting a microbiota-dependent cytotoxicity. Under normal physiological conditions, metabolites produced by gut microorganisms are not expected to interact directly with central nervous system cells; however, this may change in the presence of increased intestinal permeability, also known as “leaky gut.” Although fluoxetine does not directly target the epithelial barrier, studies have demonstrated that it alters the gut microbiota [7] suppressing the growth of certain bacterial species and thereby potentially reducing populations that contribute to epithelial integrity. Furthermore, fluoxetine may influence gut epithelial cell function by modulating serotonin levels, as serotonin plays a critical role in the regulation of tight junctions between epithelial cells [32]. Additionally, fluoxetine has been reported to increase intestinal inflammation in some individuals, which disrupts the epithelial barrier and enhances its permeability [33]. Therefore, in conditions of increased gut permeability, it is plausible that microbially altered metabolites resulting from fluoxetine exposure could enter the circulation and cross the blood-brain barrier, ultimately interacting with central nervous system cells.

When the effects of control (K24, K48, K72) and fluoxetine-exposed (M24, M48, M72) bacterial supernatants were compared at 0.5% or 1% concentration (K24 vs. M24 or K48 vs. M48 or K72 vs. M72), no statistically significant difference was found between these groups in both HMC3 and SW480 cells. Although this result shows that there is no significant difference in the effects of fluoxetine-treated and untreated bacterial supernatants on the viability values ​​of both cells, the decrease in viability was observed to be slightly more pronounced in SW480 cells treated with 0.5% M48 and SW480 cells treated with 1% M72. This observation suggests that fluoxetine exposure may alter bacterial metabolic pathways in a way that increases the toxic potential of existing compounds or leads to the formation of new bioactive molecules that have adverse effects on host cells. Although this study did not directly assess host-level outcomes, including SW480 and HMC3 cells provides preliminary insights into how supernatants affected by fluoxetine may interact with host-associated cell types. This provides preliminary information that may inform future research on drug-microbiota-host interactions.

In light of these findings, systematically investigating drug–microbiota–host interactions is critically important for ensuring drug safety, developing personalized treatment strategies, and establishing microbiota-based therapeutic approaches. Future studies that comprehensively address this tripartite interaction may enable the development of new perspectives for clinical applications. As an exploratory study, our work provides preliminary insights into fluoxetine-induced changes in bacterial physiology and their downstream cellular effects. However, the selected experimental conditions did not fully reflect physiological exposures. The exact luminal concentrations, duration of exposure in the intestinal environment, and systemic pharmacokinetics of fluoxetine remain insufficiently defined. Therefore, while our findings are exploratory, they provide a coherent experimental basis for understanding how antidepressant exposure may modulate bacterial physiology and host interactions. To better reflect in vivo conditions, future studies should integrate pharmacokinetic data with clinically relevant exposure models and analyze dose–response relationships under physiologically accurate conditions. Further investigations, including bacterial viability assays, OMV isolation, and proteomic/metabolomic analyses, are necessary to confirm these findings and clarify the underlying mechanisms in more detail.

## Data Availability

No datasets were generated or analysed during the current study.
